# Stress drives myelopoiesis to impair atherosclerosis resolution

**DOI:** 10.21203/rs.3.rs-6727599/v1

**Published:** 2025-06-09

**Authors:** Edward Fisher, Ozlem Tufanli, Bianca Scolaro, Giovanni Civieri, Florencia Schlamp, Sofie Delbare, Ada Weinstock, Flurin Cathomas, Stephanie Pena, Angélica Torres Berrío, Eric Parise, Kenny Chan, Lyonna Parise, Michael Osborne, Zahi Fayad, Eric Nestler, Filip Swirski, Ahmed Tawakol, Scott Russo

**Affiliations:** NYU Grossman School of Medicine; NYU Grossman School of Medicine; NYU Grossman School of Medicine; Massachusetts General Hospital; NYU Grossman School of Medicine; NYU Grossman School of Medicine; University of Chicago Pritzker School of Medicine; Icahn School of Medicine at Mount Sinai; NYU Grossman School of Medicine; Icahn School of Medicine at Mount Sinai; Icahn School of Medicine at Mount Sinai; Icahn School of Medicine at Mount Sinai; Icahn School of Medicine at Mount Sinai; Massachusetts General Hospital; Icahn School of Medicine at Mount Sinai; Icahn School of Medicine at Mount Sinai; Icahn School of Medicine at Mount Sinai; Harvard Medical School; Icahn School of Medicine at Mount Sinai

## Abstract

Atherosclerotic cardiovascular diseases (ASCVD) remain the leading cause of death globally. Animal and human studies link psychological stress-related disorders to ASCVD. Despite this accumulating evidence linking stress to increased cardiovascular disease (CVD) risk, it remains unclear whether stress impairs the benefits of standard risk-reduction therapies, of which lipid-lowering remains the most common, or whether this increased risk is driven by systemic inflammatory states. We tested the hypothesis that psychological stress limits the benefits of lipid lowering on resolving inflammation in atherosclerotic plaques by combining two established mouse models, namely one in which levels of atherogenic LDL cholesterol (LDL-C) can be lowered after plaques develop, and the other a model of chronic social defeat stress (CSDS). Here we show that mice susceptible to CSDS (“SUS”) had attenuated benefits of LDL-C lowering compared to control (CON) or resilient (RES) mice. Moreover, in SUS mice (vs. CON or RES) there was heightened inflammation in the plaque macrophages, with evidence that this was a result of re-programming in the bone marrow (BM) of the precursors of macrophages, namely monocytes. Remarkably, these observations aligned with human imaging studies, in which LDL-C lowering therapy was not as effective in reducing either systemic or arterial inflammation in subjects with higher (vs. lower) neural imaging measures of psychological stress. In summary, the integration of the mouse model and human data provides important mechanistic and clinical insights into the crucial role of chronic stress in ASCVD, highlighting that this common risk factor impairs the anti-atherosclerotic benefits of lipid-lowering medications and may represent an important determinant of residual ASCVD risk.

## INTRODUCTION

Atherosclerotic cardiovascular diseases (ASCVD) remain the leading cause of death globally^1^. Psychological stress, which can precipitate depression, anxiety and PTSD, has been extensively studied in both clinical and experimental settings as a significant prognostic factor for CVD, particularly among populations at high risk from other factors^1,2^. Moreover, high levels of perceived psychological stress following myocardial infarction (MI) substantially elevates the risk of death by 40%^3^. Despite accumulating evidence linking stress to increased CVD risk, it’s unclear whether stress impairs the benefits of standard risk-reduction therapies, of which lipid-lowering remains the most common^4^. Despite an effective reduction of hypercholesterolemia following treatment with statins, systemic inflammation and a high plaque burden persist in a significant proportion of patients, resulting in a relatively high residual risk for ASCVD^5–7^.

Recent studies in both mice and humans have indicated that stress can trigger inflammation and worsen atherosclerosis^8–10,11^. For example, in mice, enhanced myeloid progenitor cell proliferation in the bone marrow and reprogramming of monocytes to a proinflammatory phenotype have been found after exposure to chronic variable stressors such as crowding, isolation, cage tilt and changes in bedding^8,12^. These data are consistent with other studies showing that psychological stress increases myeloid cell production^13,14^. In addition, hyperlipidemic *Apoe*^−/−^ mice had increased atherosclerosis with higher macrophage plaque content after exposure to variable stressors^8^. The clinical relevance of these findings is supported by cross- species studies (i.e., rodent and human) linking the increased activity of the amygdala—a brain region activated by stressful experiences and anxiety—with several indices of CVD risk, including increased bone marrow activity, circulating inflammatory markers, and arterial inflammation^11,15–19^.

Based on these studies, we hypothesized that psychological stress compromises the benefits of lipid-lowering therapies in humans and mice. Using the chronic social defeat stress (CSDS) model in mice, we previously showed that stress causes immunological and behavioral alterations similar to that observed in human subjects^20–22^. Another valuable feature of this model is that after exposure to CSDS, mice exhibit individual differences (i.e., susceptible vs. resilient), like human subjects, across various immunological and cardiovascular endpoints. Thus, we utilized the CSDS model to explore individual differences in how psychological stress contributes to the impairment of lipid- lowering treatments in plaque resolution.

As will be presented, our findings reveal that stress susceptibility, but not resilience, is associated with significant impairments in the resolution of systemic and plaque inflammation following lipid-lowering treatment. Notably, these findings align with human imaging studies, in which statin therapy is less effective in reducing either systemic or vascular inflammation in subjects with higher neural imaging measures of psychological stress. Using single-cell RNA-seq combined with flow cytometry, we show that CSDS induces distinct changes in the distribution and characteristics of monocyte cell subpopulations in the bone marrow, with stress susceptibility resulting in an increase in inflammatory monocytes. Furthermore, bone marrow transplant studies provide additional evidence that stress-susceptible bone marrow cells retain an inflammatory memory when transferred to naïve mice, thereby intensifying the detrimental effects of high cholesterol levels on atherosclerosis development.

## RESULTS

### CSDS impairs atherosclerosis regression in susceptible, but not resilient, mice

**Figure 1:**
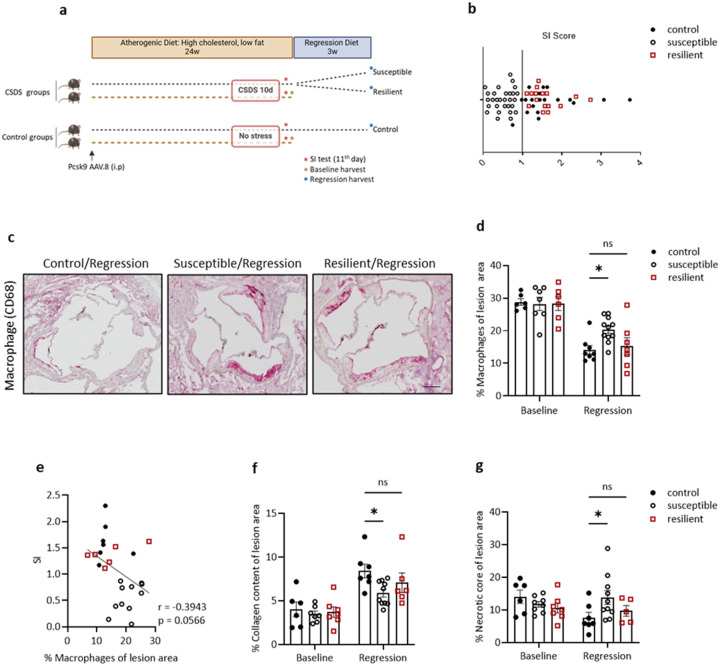
Susceptibility to CSDS, but not resilience, impairs plaque regression despite the reversal of dyslipidemia. **a.** Experimental outline: Atherosclerosis was established in C57BL6/J mice by injection of AAV.8-PCSK9 followed by atherogenic diet feeding for 24 weeks (Baseline). CSDS (10 days) was applied at the end of the baseline. Mice were categorized based on social interaction (SI) tests as SUS and RES. Control (CON) mice were kept under the same conditions but were not exposed to aggressor mice. After the SI testing, all mice were kept under a regression diet, including MTP inhibitor, for 3 weeks. **b.** SI score of mice. Stress-resilient (RES; red squares), stress-susceptible (SUS; black unfilled circles), and control (CON; black filled circles) groups were shown (n=14,26,20 each group). **c.d.** Representative images and quantification of aortic plaques after CD68 staining. One-way ANOVA. Scale bar: 100 μm **e.** Pearson correlation between SI score and CD68 %. **f.** Quantification of plaque collagen and **g.** necrotic core area after picrosirius red staining (n=5–10 each group). One-way ANOVA. Data are the mean ± SEM. P values adjusted for multiple comparisons. *P < 0.05.

The creation of mouse models for reversing atherosclerosis has facilitated the identification of modifiable factors and pathways, as well as techniques to reduce lipid levels, the most common approach to lowering the risk of CVD in patients^23,24^. Here we first established atherosclerosis in C57BL6/J mice by using adeno-associated virus (AAV.8) to overexpress proprotein convertase subtilisin/kexin type 9 (*Pcsk9*) in the liver, resulting in low-density lipoprotein receptor (LDLR)-deficiency, in combination with feeding the mice an atherogenic diet^25,26^. After 24 weeks, by which time advanced plaques formed, mice were switched to a microsomal triglyceride transfer protein (MTP) inhibitor-supplemented diet to induce cholesterol lowering and atherosclerosis regression, as we have done before^23,27^. This experimental design is summarized in Figure 1a.

To investigate the effects of psychosocial stress on plaque regression, we used the CSDS paradigm^28,29^. This protocol induces subordination of experimental C57BL6/J mice by aggressive CD-1 mice through the combination of physical and sensory contact over 10 days. Despite being exposed to the same stressor, mice exhibit individual differences in their responses. Based on social interaction (SI) scores^30^, most of the mice show social avoidance (as reflected by lower SI scores) and are classified as susceptible (SUS). In comparison a subset has higher SI scores and behaves similarly to unstressed control (CON) mice. These mice are classified as resilient (RES). We conducted CSDS at the end of the atherogenic diet period to test the hypothesis that psychological stress would interfere with the benefits of lipid-lowering on plaques. Before switching to the regression treatment period (i.e., the MTP inhibitor-supplemented diet), mice were categorized as SUS and RES based on their SI scores (Figure 1b).

We observed major reductions of cholesterol levels after regression treatment in all groups of mice (**Extended Data Figure 1a**). We found no changes in body or tissue weights (i.e., liver, brown adipose tissue [Bat]), except increased spleen size in SUS compared to CON mice (**Extended Data Figures 1b-e**). Notably, though the plaque areas were similar in the 3 regression groups, there were a number of compositional features that varied. Using CD68 as a commonly accepted marker of plaque macrophages^31^, SUS mice had higher CD68^+^ abundance than CON or RES mice (Figures 1c, d, **Extended Data Figures 2a, b**). We also found a negative correlation between SI score and % CD68^+^ cells in plaques (Figure 1e), strongly suggesting that stress is a significant factor in the responses of macrophages in plaques to lipid lowering. We also observed that SUS mice had less plaque collagen and larger necrotic core areas—indices of plaque instability in humans—compared to CON or RES mice (Figures 1f, g, **Extended Data Figure 2c**), thereby explaining the overall similarity in plaque size in the three groups despite the difference in CD68^+^ cell contents. Clinical studies have long established that plaque composition (particularly its macrophage content), and not size, determines the risk of myocardial infarction^32^. These results suggest that the effect of stress on maintaining a higher macrophage content in plaques after lipid reduction contributes to the increased CVD risk associated with stress and depression.

### CSDS affects the kinetics and molecular features of myeloid cells

To determine the mechanisms by which plaque macrophages are increased in SUS mice, we 1) quantified circulating monocytes (the precursors of macrophages) and their subtypes (Ly6C^hi^, Ly6C^lo^), 2) analyzed *in vivo* monocyte-macrophage plaque kinetics, and 3) assessed plaque macrophages at the molecular level by laser capture microscopy and RNAseq.

**Figure 2:**
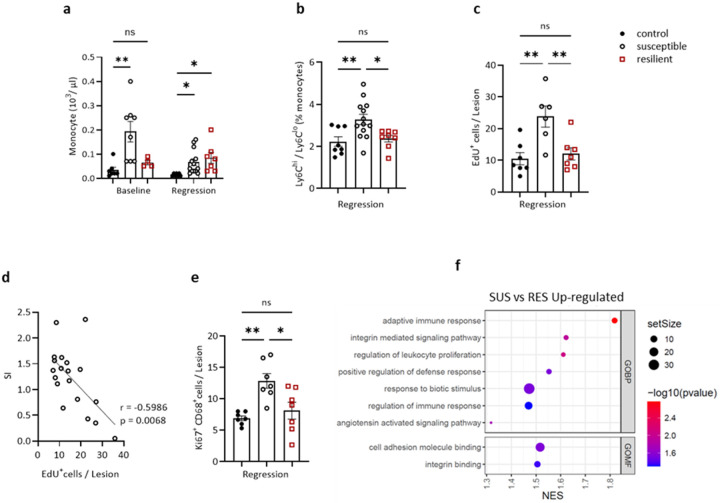
Susceptibility to CSDS increases monocyte numbers in circulation and alters monocyte/macrophage dynamics and features in regressing plaques. **a.** Number of monocytes in the circulation of CON, SUS, RES mice at baseline and after 3 weeks regression diet. One-way ANOVA. **b.** Analysis of Ly6C^hi^/Ly6C^lo^ monocyte proportion via flow cytometry after 3 weeks on a regression diet. One-way ANOVA. **c.** Quantifying of the recruitment of EdU-labeled Ly6C^hi^ monocytes to aortic root plaques of mice after 3 weeks regression diet (n=6–7 each group). One-way ANOVA. **d.** Pearson correlation between SI score and EdU-labeled Ly6C^hi^ monocyte recruitment to plaques. **e.** Quantification of immunostaining for the cellular proliferation marker Ki67 (n=6–7 for each group). One-way ANOVA. **f.** Gene ontology (GO) terms of significantly (p-value<0.05) upregulated genes of plaque macrophages compared between SUS vs RES mice. Plaque macrophages were isolated by laser capture microdissection, and transcripts were analyzed using bulk RNA sequencing. Data are the mean ± SEM. P values adjusted for multiple comparisons. *P < 0.05, **P < 0.01

As shown in Figure 2a, at baseline (i.e., at the end of the atherosclerosis progression period) there was a significant increase in total monocytes in the SUS group. In the regression groups, circulating monocytes were lowest in CON mice (Figure 2a). Within the monocyte population, Ly6C^hi^ cells are thought to have greater inflammatory potential compared to Ly6C^lo^ cells^33^. It is noteworthy, then, that in the regression groups, the ratio of Ly6C^hi^ to Ly6C^lo^ monocytes was highest in the SUS group (Figure 2b, **Extended Data Figure 3b**). Turning to neutrophils, which are the other major circulating innate immune cells, their circulating levels were elevated in the two groups exposed to stress (i.e., SUS, RES), but were at similar levels among the three groups in the regression period (**Extended Data Figure 3a**).

Next, we assessed the effects of CSDS on monocyte recruitment into aortic root plaques. To measure monocyte recruitment, circulating Ly6C^hi^ monocytes were pulse-labeled 72 h before the end of the regression treatment with 5-ethynyl-2′-deoxyuridine (EdU) using a protocol we have previously established for kinetic studies of monocyte *in vivo* trafficking^34^. As shown in Figure 2c and **Extended Data Figure 4a**, during the regression period, there was a significant increase in monocyte recruitment into plaques of SUS compared to CON and RES mice. Notably, as we saw with the correlation of SI with plaque CD68^+^%, there was also an inverse relationship between SI score and monocyte recruitment (Figure 2d). We also examined whether the proliferation of plaque macrophages (a phenomenon reported before^35^) contributed to the differences in plaque CD68^+^ cell content in the regression groups. Thus, we quantified the staining for the cellular proliferation marker Ki67. Compared to CON and RES, we observed increased proliferation of macrophages in SUS mice (Figure 2e, **Extended Data Figure 4b**). These data suggest that the kinetic basis for greater content of plaque macrophages in SUS mice compared to CON or RES mice includes increased monocyte recruitment and macrophage proliferation.

The above studies were primarily focused on the plaque content of macrophages, but another important aspect is their phenotype, which can span a wide spectrum from inflammation to its resolution. To approach this at the molecular level, RNA was isolated and sequenced from plaque macrophages that were selected by laser-capture microscopy (LCM)^36^. We then investigated differences in the transcriptome of plaque macrophages from CON, SUS, and RES mice at the end of the regression period.

Changes in plaque macrophage gene expression were most pronounced in SUS mice, with a total of 551 upregulated differentially expressed genes (DEGs) vs CON mice. Additionally, there were 358 upregulated DEGs in RES vs CON mice, and 480 upregulated DEGs in SUS vs RES (p-val < 0.05). Consistent with data in Figures 1d–g, principal component analysis (PCA) on significantly upregulated DEGs (p-val < 0.05) showed the greatest variance between SUS mice plaque macrophages compared to CON and RES mice, which clustered closely together (**Extended Data Figure 5a**). We then performed gene ontology (GO) enrichment analysis of biological processes and molecular functions (Figure 2f). Genes upregulated in SUS vs RES mice were involved in GO biological processes (GOBP) such as adaptive immune response, integrin mediated signaling pathways, positive regulation of leukocyte proliferation, defense response and immune response. In GO molecular functions (GOMF), upregulated processes included cell adhesion molecule binding and integrin binding.

These data begin to form a framework for understanding why SUS mice have an impaired response to atherosclerosis regression-inducing lipid lowering. One basis for the increase in plaque macrophage burden compared to CON and RES mice is the combination of both an increase in the pool of circulating monocytes and their increased trafficking to plaques. This is further abetted by activation of cell adhesion (based on the bioinformatic analysis) and the local proliferation of plaque macrophages (based on bioinformatics and Ki67 staining). In addition, there is activation of immune pathways in plaque macrophages, and enrichment in Ly6C^hi^ circulating monocytes, both of which are consistent with a higher inflammatory state. Based on these results, we hypothesized that CSDS may affect monocytes prior to their release from BM into the circulation, which we tested in the following sections.

### CSDS affects distribution and molecular characteristics of myeloid cells in bone marrow

**Figure 3:**
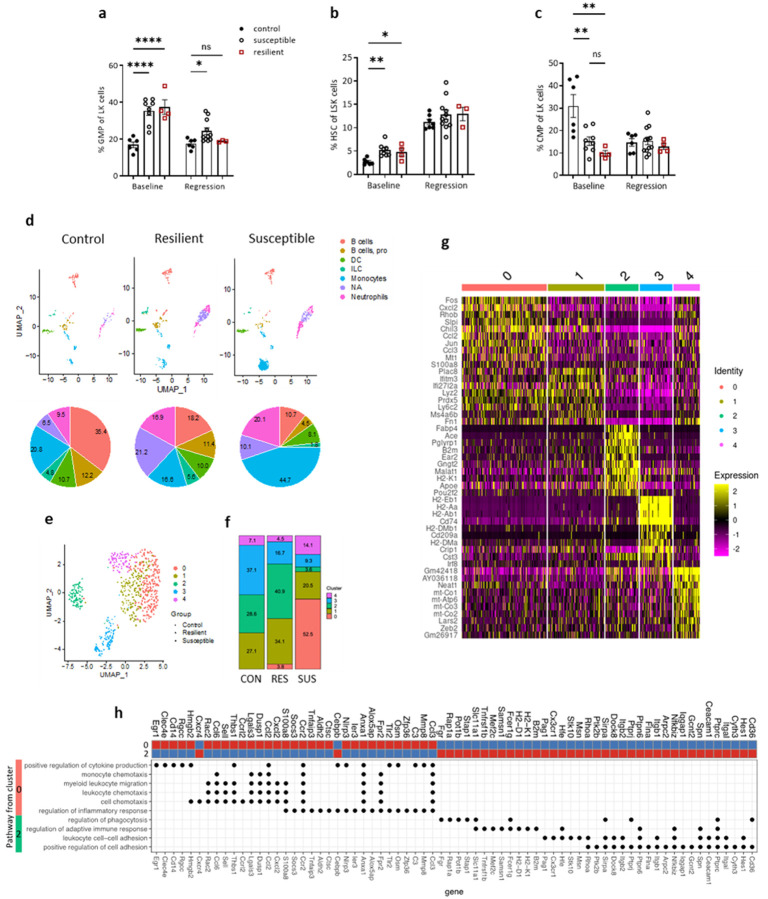
Susceptibility and resilience to CSDS increase progenitor cells and induce distinct changes in the distribution and characteristics of monocyte cell subpopulations in the bone marrow. Levels of **a.** granulocyte- macrophage progenitors (GMPs[%Lin-/Kit+/Sca1-]), **b.** progenitor cells (HSCs[%LSK]), **c.** common myeloid progenitors (CMP [%Lin-/Kit+/Sca1-]) in the bone marrow of stressed and control mice. **d.** Uniform Manifold Approximation and Projection (UMAP) representation of CD45^+^ cells identified by scRNAseq of mouse bone marrow with pie chart representation of relative (%) abundance of cells per annotated population in each treatment. **e.** UMAP representation of 5 monocyte sub-clusters and **f,** bar plot of relative (%) abundance of cells per monocyte sub-cluster in CON, SUS, and RES mice. **g.** Heatmap of top 10 cluster-defining protein-coding genes. **h.** Heatmap of monocyte clusters ‘0’ and ‘2’ Z-scores of normalized mRNA expression values for genes found in the top enriched pathways in each monocyte cluster. Dots below the heatmap indicate gene pathway assignment. Data are the mean ± SEM. P values adjusted for one-way ANOVA multiple comparisons. *P < 0.05, **P < 0.01, ***P < 0.001, ****P < 0.0001.

First, we investigated the impact of stress on hematopoiesis and the subsequent immune cell profile in bone marrow (BM). As previously described^8,12^, we found hematopoiesis activity in both SUS and RES mice compared to CON mice. This was evidenced by elevated levels of granulocyte-monocyte progenitors (GMP) and hematopoietic stem cells (HSC) (Figures 3a, b). Conversely, there was a decrease in the common myeloid progenitor (CMP) population in the bone marrow at baseline, showing a high commitment of CMP compared to GMP (Figure 3c). We observed a reduction in GMP cells during regression treatment in both stressed groups (i.e., SUS and RES) compared to baseline, indicating that aggressive lipid reduction reduces GMP cell production while increasing HSCs in all groups, which would serve to balance progenitor cell storage (Figures 3a, b, c, **Extended Data Figure 3c**).

To assess if alterations in progenitor cell numbers were accompanied by molecular changes in immune cell populations after CSDS, we isolated CD45^+^ leukocytes from the BM of stressed (SUS, RES) and non-stressed (CON) mice, and profiled their transcriptomes using single-cell RNA sequencing (scRNAseq; **Extended Data Figure 6a**). CSDS led to an increase in the proportion of neutrophils and a decrease in B cells in both RES and SUS mice compared to CON mice, in line with the results for circulating cells (**Extended Data Figure 3a** and data not shown). Notably, BM monocytes had the largest percentage increase among all the immune cells in SUS group (Figure 3d).

We performed differential gene expression analysis of the BM monocyte population (**Extended Data Figures 6b, d, f**). GO and KEGG pathway enrichment analyses were then performed with the DEGs. DEGs in SUS vs CON mice (289 DEGs, p-value< 0.05) were associated with upregulated GO biological processes, including regulation of NIK/NF kappaB signaling, endothelial cell migration, response to chemokine, response to Interleukin 1, inflammatory processes, macrophage chemotaxis, response to endoplasmic reticulum stress, positive regulation of cell death, and GO molecular processes including function of cytokine and chemokine receptor binding activities (**Extended Data Figure 6c**). Analysis of SUS vs RES DEGs (180 DEGs, p-value < 0.05) showed upregulated GO biological processes, including monocyte activation, response to endoplasmic reticulum stress and integrated stress response signaling, response to interleukin 1 and cytokine and chemokine receptor binding activities, and GO molecular processes including cytokine and chemokine receptor binding activities (**Extended Data Figure 6e**). In contrast to these comparisons, we observed a few differentially expressed genes (40 DEGs, p-value < 0.05) when we compared RES vs. CON mice. Furthermore, RES monocytes showed more similarity with the CON monocyte transcriptome compared with SUS mice (**Extended Data Figure 6f**). Both findings are consistent with lower plaque macrophage content in the RES and CON relative to SUS groups. CSDS also induced cell migration and the integrated stress response (ISR) pathway through activation of eIF2a-Atf3-Chop in SUS monocytes (**Extended Data Figures 6d, e**). In addition, we found that SUS BM monocytes had a significantly greater inflammatory signature than BM monocytes in CON and RES mice (**Extended Data Figure 6g**). Taken together, the data presented so far show that CSDS increases both the percentage of monocytes in BM as well as their inflammatory and stress signatures in SUS mice compared to CON and RES mice. This was despite the fact that both groups exposed to stress (SUS and RES) exhibited similar increases in the GMP population in BM, suggesting that exposure to stress, in addition to effects on monocyte precursor proliferation, also exerts effects on their differentiation bias.

To further explore the molecular diversity of BM-monocytes in the regression groups, we performed sub-cluster analysis of the monocyte population using Seurat. This revealed five distinct sub-clusters based on their transcriptional profiles (Figure 3e). Cluster 0 was dramatically enriched in SUS mice compared to CON and RES mice (52.5% in SUS vs. 3.8% in RES), while cluster 2 was much less represented in SUS vs RES (3.6% vs. 40.9%; Figure 3f). To determine cluster-defining genes, we conducted a DEG analysis (Figure 3g). Several genes associated with inflammatory and migratory processes were found to be upregulated in Cluster 0; e.g., *Fos* (known to have higher expression in classical, inflammation-prone monocytes) and *Jun*, which together with *Fos* form activator protein 1 (AP-1) (FOS/JUN)^37,38^, and the chemokines; *Cxcl2, Ccl2* (which have a crucial role in monocyte chemotaxis^39^ and egress from bone marrow^40^) and *Ccl3*. Consistent with the DEGs, GO term analysis of upregulated genes in cluster 0 revealed associations with pathways such as leukocyte migration and chemotaxis, regulation of inflammatory response and cytokine production (Figure 3h). The DEGs in cluster 2 included *Fabp4, Ace, Ear2, ApoE*, which are associated with non-classical monocytes^41,42^ (i.e., cells that are thought to be precursors of inflammation-resolving macrophages^43,44^). GO pathway analysis of upregulated genes in Cluster 2 revealed associations with leukocyte cell-cell adhesion, adaptive immune response and regulation of phagocytosis (Figure 3h). This latter process is fundamental to efferocytosis, an inflammation-resolving mechanism that limits the necrotic core^45^ and, therefore, is consistent with the data in Figure 1g. Ly6C is a marker encoded by *Ly6c2* whose expression level is commonly used to differentiate between classical (high) and non-classical (low) monocytes. Notably, relative to the other clusters, the *Ly6c2* gene had higher and lower expression, respectively, in Cluster 0 and Cluster 2 (**Extended Data Figures 7a, b**).

We then investigated the similarities of the monocyte sub-clusters with published RNA-seq data of Ly6C^hi^ and Ly6C^lo^ BM cells, using the data from Mildner *et al*.^46^. Based on these comparisons, Clusters 0 and 1 (high expressors of *Ly6c2*) showed similarity to the Ly6C^hi^ monocyte gene expression patterns, based on the high expression of *Sell, Id1, Ccr2, Lgals3, Trem2, Lyz2* genes, and low expression of *ApoE, Cd36, Cd74, Cx3cr1, Fcgr4, H2-Aa, H2-Ab1, Nr4a1* genes. Also based on this published data^46^, the pattern of gene expression in Clusters 2 and 3 (upregulated genes *ApoE, Cd36, Cd74, Cx3cr1, Fcgr4, H2-Aa, H2-Ab1, Nr4a1*) resembled that of Ly6C^lo^ monocytes (**Extended Data Figure 7c**). Although *Ly6c2, Ccr2, and Cx3cr1* were expressed across the monocyte sub-clusters, there were significant differences in their expression (**Extended Data Figures 7 b, f, h**). Based on this, manual annotation supported that classical monocytes (higher expression levels of *Ly6c2* and *Ccr2* in Cluster 1 and 0 vs. Cluster 2) were more enriched in SUS mice, while non-classical monocytes (expression levels of *Ly6c2* lower and *Cx3cr1* higher in Cluster 2 vs. other clusters) were more enriched in RES mice (**Extended Data Figures 7d-h**).

These data indicate that the BM compartment responds strongly and differentially in SUS and RES mice to CSDS based on proportions of monocyte sub-populations and their patterns of gene expression. In general, the results are consistent with the differential levels in circulating monocytes (Figure 2b) found in SUS and RES mice at the end of the stress protocol, and in the plaque properties (including macrophage content) after lipid reduction (Figures 1, 2). Overall, CSDS shifts monocytes towards a more inflammatory phenotype in SUS mice, evident in monocyte cluster 0, while shifting them towards a more non-classical phenotype in RES mice, represented by cluster 2.

### CSDS effects on transcription factors and gene regulatory networks in monocytes

#### Bone marrow studies:

We next sought to identify transcription factors that underly the differences summarized in the previous section in the BM monocyte gene expression patterns in SUS, RES, and CON mice after the regression treatment period. Thus, we performed single-cell transcription factor regulatory network inference using pySCENIC^47^. pySCENIC calculates activity of regulons, i.e. transcription factors and their predicted target genes, based on gene expression patterns and enrichment of transcription factor binding motifs^47^. Clustering of cells based on pySCENIC regulon activity can determine monocyte subpopulations based on their distinct sets of active transcription factors (**Extended Data Figure 8a**). Regulons for the transcription factors JUN, JUNB, FOS, FOSB, ATF3 AND EGR2 had higher activity scores in BM monocytes from SUS mice compared to those from both RES and CON mice (**Extended Data Figures 8b, c**). These transcription factors are predicted to drive the upregulation of inflammatory genes associated with immediate (early) responses to cell-extrinsic and cell-intrinsic signals, such as immune responses or cellular stress^48,49^. The increase in regulon activity of immediate early transcription factors in SUS mice is consistent with the finding of increased immune and integrated stress responses in BM monocytes and plaque macrophages in SUS mice (**Extended Data Figures 6b, d**).

Regulon activity for the transcription factors NFIC, STAT1, BACH and ETV3 was downregulated in SUS vs CON mice, whereas regulon activity for IKZF1, SP1, MEF2A, HES1, and SRF was downregulated in SUS vs RES mice (**Extended Data Figures 8b, c**). Most of these factors are associated with conversion of classical Ly6C^hi^ to non-classical Ly6C^lo^ monocytes (NOTCH regulated HES1 and SP1 and IKZF1 and IKZF3^50,51^) and with cell adhesion (MEF2A)^52^. These data are consistent with the enrichment of Cluster 2 (non-classical like monocytes) in RES and CON mice compared to SUS mice. Activities of the most significantly upregulated regulons for JUN, FOS, ATF3, and downregulated regulons for MEF2A and HES1 are shown in **Extended Data Figures 8d-h**. Due to the importance of the NOTCH2-HES1 axis in the control of the conversion of Ly6c^hi^ to Ly6c^lo^ monocytes^51,53^, we examined gene expression patterns of HES1 target genes identified by pySCENIC in monocytes in all 3 groups (i.e., SUS, RES and CON). The gene expression profiling of BM monocytes indicated a reduction in the expression of genes linked to HES1 signaling in the SUS group and an increase in the RES and CON groups (**Extended Data Figures 8i**). These data are consistent with the heightened inflammatory phenotype of monocytes in SUS vs. CON or RES mice.

#### Comparisons to circulating monocytes in CSDS mice:

To determine whether the results in BM monocytes were relevant to circulating monocytes, we compared our single-cell RNA sequencing results with published data sets of circulating Ly6C^hi^ monocyte-specific bulk RNA sequencing results (Cathomas *et al*.^20^) from CSDS-exposed mice (**Extended Data Figure 9a**). Analysis of the DEGS in the SUS and RES mice from these two datasets revealed 395 shared monocyte DEGs in response to CSDS. Of these 395 DEGs, there were 146 upregulated and 81 downregulated transcripts with common directionality of expression in both datasets. Pathway analysis of the commonly upregulated genes revealed enrichment in the inflammatory response, cell chemotaxis, leukocyte migration, and chemotaxis (**Extended Data Figure 9b**). These data suggested that changes in the BM monocyte transcriptome related to activation of chemotaxis, cell migration, and regulation of inflammatory pathways are retained in the monocytes released into the circulation regardless of ASCVD status.

### Adverse effects of CSDS on atherosclerosis are transmissible by bone marrow transplantation

**Figure 4:**
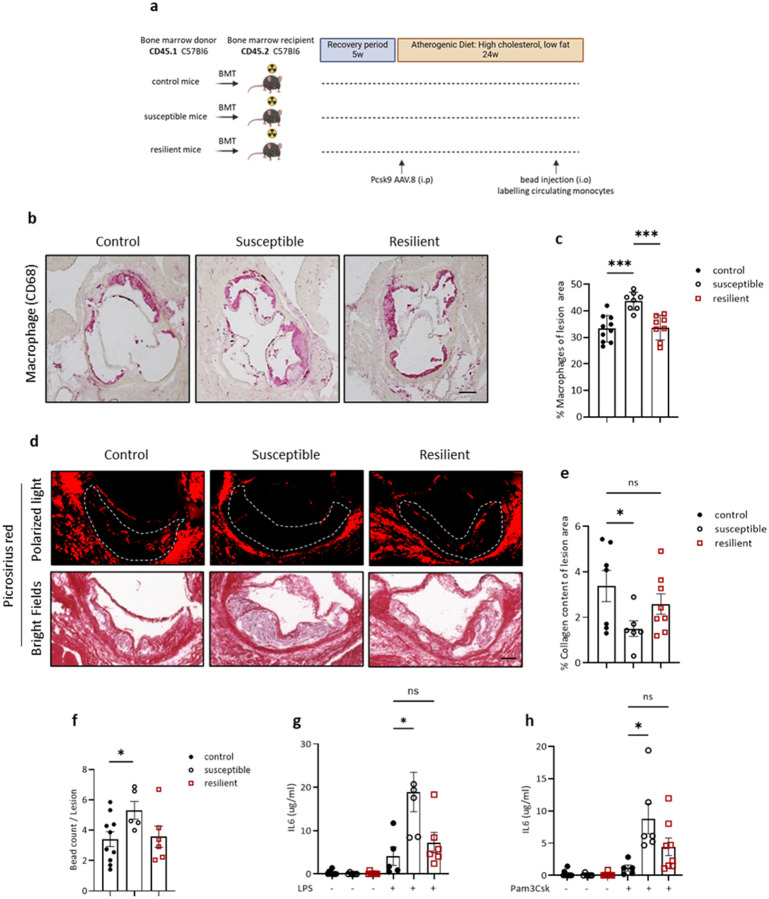
BM transplantation from SUS, CON, and RES mice into naïve recipients results in differences in atherosclerosis progression. **a.** Outline of bone marrow chimera experiment, with bone marrow transplantation (BMT) from wild type CD45.1 C56Bl6 CON, SUS and RES mice to wild type CD45.2 C56Bl6 mice on day 0, followed by 4 weeks of recovery and PCSK9 AAV.8 injection. All mice were then kept on an atherogenic diet for 24 weeks. **b, c.** Representative images and quantification of immunohistochemical staining for the macrophage marker CD68 (red) in aortic root plaques of mice (n=10,8,8 for CON, SUS and RES mice respectively). One-way ANOVA. **d. e.** Picrosirius red staining to identify collagen area using the bright field (bottom panels) or polarized light (top panels) (n=7,6,8 for CON, SUS, and RES, respectively). One-way ANOVA. **f.** Quantification of the recruitment of bead-labelled monocytes to aortic plaques of mice (n=10,5,6 for CON, SUS, and RES, respectively). *P < 0.05, two-tailed Student’s T-test. **g.** Lipopolysaccharide (LPS) (10 ng/ml 16 hr.) and **h.**PAM3CSK stimulated (10 ng/ml 16 hr) IL-6 response of bone marrow cells isolated from SUS, RES or CON mice. One-way ANOVA. Data are the mean ± SEM. P values adjusted for one-way ANOVA multiple comparisons. *P < 0.05, **P < 0.01, ***P < 0.001.

The transcriptomic and *in vivo* data have shown that monocyte inflammatory phenotypes at BM and circulation levels were highest in SUS mice. Current thinking suggests that this may represent epigenetic re-programming of these macrophage precursors, which can result in durable effects on responses to inflammatory stimuli^54^. A standard approach to testing this possibility is to transfer bone marrow hematopoietic stem cells from one mouse to another and then determine the fidelity of the disease response. In the case of atherosclerosis, we reconstituted the BM of irradiated stress naïve C57BL6/J (CD45.2^+^) recipient mice by transplantation of BM from SUS, RES, and CON C57BL6/J (CD45.1^+^) mice. The use of CD45.1 and CD45.2 mice allowed us to confirm the efficiency of BM transplantation in reconstituting the immune cells of the recipients. After a 4-week recovery period to allow for complete reconstitution by donor hematopoietic cells, mice were injected with PCSK9 AAV.8 to cause hepatic LDLr-deficiency and hypercholesterolemia. The recipient mice were then fed the atherogenic diet for 24 weeks but were not exposed to CSDS (Figure 4a).

Transplantation of SUS BM cells worsened plaque inflammation as reflected by a higher content of plaque macrophages (Figures 4b, c) and a relative decrease in plaque collagen (Figures 4d, e; as noted before, one of the indices of plaque stability in human plaques). Both results are consistent with the adverse effects we observed in the regression study (Figures 1d, f). These results could not be explained by differences in the efficiency of the BM transplantations, as we confirmed ~90% donor leukocyte chimerism in all the recipients and found no differences in proportions of donor-derived leukocytes between SUS, RES and CON BM chimeras (**Extended Data Figure 10a**).

Next, we assessed monocyte recruitment into aortic root plaques as described earlier^55^ We observed significantly more monocyte recruitment into plaques in the recipient mice transplanted with SUS BM (Figure 4f), again consistent with the results from the regression study (Figure 2c). To assess whether monocytes from SUS mice were also more reactive to stimulation, BM cells were isolated from SUS, RES and CON mice and then stimulated with the Toll-like receptor (TLR) 4 agonist, lipopolysaccharide (LPS), and the Toll-like receptor 2 agonist, Pam3Csk4, for 16 h and the secretion of cytokine IL-6 was assessed. Notably, BM cells from recipient mice transplanted with SUS BM produced higher levels of IL-6 in response to TLR agonists compared to RES and CON BM recipients (Figures 4g, h). These data strongly suggest that BM myeloid cells underwent programming in SUS mice that was sustained in the transplant recipients, such that higher inflammation was observed at both the *in vivo* and *ex vivo* levels.

### Vascular and systemic inflammation after lipid lowering is attenuated in stressed human subjects

The present results in the aggregate have shown that stress susceptibility in mice impaired the benefits of lipid-lowering on atherosclerotic plaques. To establish the clinical relevance of this finding, we analyzed arterial inflammation in human subjects using imaging^15^ and blood levels of high-sensitivity C-reactive protein (hs-CRP, a marker of inflammation and cardiovascular risk)^56^. The subjects were divided into groups based on stress-associated neural activity measured using ^18^F-fluorodeoxyglucose positron emission tomography/computed tomography (PET/CT) imaging. Accordingly, the well-validated measure of amygdala activity divided by regulatory activity of the cortex (AmygA_C_) was derived. It was previously shown that AmygA_C_ is associated with chronic stress exposure, psychometric measures of stress, and higher risk of cardiovascular events^19,57^. Furthermore, neurobiological resilience (defined as lower AmygA_C_ despite stress exposures) was shown to protect against adverse cardiovascular events through decreased bone marrow activity and arterial inflammation^57^, consistent with the improved atherosclerosis regression and reduced inflammatory BM monocytes in the RES mice.

**Figure 5:**
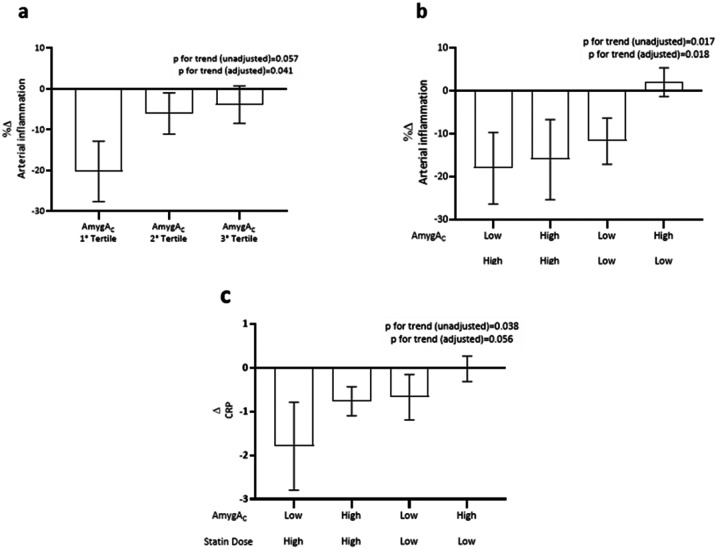
Stress-related neural activity reduces the efficacy of lipid-lowering statin therapy to reduce vascular inflammation in humans. **a.** Arterial inflammation was measured at baseline and 12-week follow-up statin treatment (p for trend 0.041). **b.** Study population is divided into 4 groups according to AmygA_C_ at baseline (low=below median vs. high=above median) and statin dose (high=80mg vs. low=10mg) (p for trend 0.018). **c.** High sensitivity C-reactive protein (hs-CRP) was measured at baseline and 12-week follow-up (p for trend 0.056). Statistical analyses were performed using the Statistical Package for Social Sciences (SPSS; version 28, IBM Corporation, Armonk, NY, USA). Data were presented as mean and standard deviation (SD) if normally distributed or as the median and interquartile range (IQR) if skewed. The trend analysis is adjusted for age, sex, and previous statin therapy (See Details in Methods). Abbreviations: AmygA_C_: amygdala to temporal lobe activity ratio; hs-CRP: high sensitivity C-reactive protein.

Using a double-blinded study design, 40 subjects with- or at a higher risk for ASCVD were randomized to a low (10 mg/daily) or high (80 mg/daily) dose of atorvastatin (ATV) in a trial assessing the effects of statins on arterial inflammation. Overall, 23 were assigned to ATV10 and 17 to ATV80. Baseline characteristics of the patients are reported in **Extended Data Table 1**. Notably, there was no significant relationship between baseline AmygA_C_ and achieved reductions in LDL after statin therapy (p=0.37). However, AmygA_C_ at baseline significantly predicted the reduction in arterial inflammation at follow-up, even after adjustment for prespecified confounders, with lower AmygA_C_ associating with more significant reductions in arterial inflammation (**Extended Data Table 2**, Figures 5a). When subjects were divided into 4 groups according to baseline AmygA_C_ (below vs above median) and statin dose (high vs. low dose ATV), subjects with low AmygA_C_ on a high statin dose experienced the most significant reduction in arterial inflammation. In contrast, patients with high AmygA_C_ on a low statin dose experienced a modest increase in arterial inflammation (Figure 5b). In a secondary analysis, we also assessed the associations between AmygA_C_ and reduction of systemic inflammation. In this analysis, we observed that AmygA_C_ and statin doses predicted reductions of hs-CRP levels in a pattern similar to what was observed for arterial inflammation (Figure 5c).

From the clinical perspective, these findings indicate that higher stress-associated neural activity (i.e., high AmygA_C_) reduces the impact of lipid-lowering statin therapy on vascular and systemic inflammation, as assessed by imaging and blood hs-CRP levels. Notably, these human data align remarkably well with the murine data. For example, despite lipid lowering in all mouse regression groups, the reduction in arterial inflammation (assessed by monocyte recruitment, plaque macrophage content and activation) was significantly attenuated in SUS mice. Similarly, the subjects with low AmygA_C_, taken to reflect resilience, had relatively more reduction in vascular inflammation, consistent with the results in the RES mice.

## DISCUSSION

Despite effective cholesterol-lowering medications, the mortality rate is still high within a group of CVD subjects due to residual inflammation^7^. Although several studies have linked chronic stress exposure to arterial inflammation, we show that chronic stress compromises the benefits of lipid- lowering therapies in humans and mice by increasing systemic and arterial inflammation. To test causality, we used a well-established mouse model of psychosocial stress, which can recapitulate many immunological and behavioral features associated with psychological stress in humans. Our results demonstrated for the first time that susceptibility to stress impaired atherosclerosis regression after lipid-lowering was related to increases in circulating monocytes and the plaque macrophages derived from them and decreases in plaque stability indices. While these findings are consistent with other forms of chronic stress on atherosclerosis progression^8,12,58,59^, this is the first report on its deleterious effects on plaque regression. Interestingly, we showed that mice that were resilient to stress—i.e., mice maintaining normal psychological function despite similar levels of stress exposure—exhibited beneficial lipid-lowering effects on cardiovascular endpoints, like unstressed CON mice.

Mechanistic studies provided evidence that monocytes in the bone marrow adopt a more inflammatory phenotype by activating both inflammatory and integrated stress pathways. This results in more inflammatory monocytes in circulation and plaques in SUS mice, not RES and CON mice, even though we find similarly elevated levels of granulocyte-monocyte progenitors (GMP) and hematopoietic stem cells (HSC) in both stressed groups. This may suggest that there are changes to the stem/progenitor pool that push monocytes to adopt a more inflammatory signature. In support of this hypothesis, transplantation of BM-derived hematopoietic progenitor cells from susceptible donors has been shown to promote stress susceptibility following social defeat stress compared to those receiving transplantation from CON donors^60^. However, whether BM from SUS conditions affects other immunological parameters and further atherosclerosis in mice was unknown. Here we showed that BM-derived hematopoietic progenitor cells from susceptible donors maintain their inflammatory phenotype weeks after transplantation, thereby influencing atherosclerosis in recipient mice. Though speculative, epigenetic changes elicited by stressful experiences may have durable effects that can be maintained even after BM transplantation resulting in increased inflammation in plaque macrophages. Our clinical data also aligns remarkably well with these murine data. Hence, our study in mice supports investigation into strategies to enhance the therapeutic efficacy of lipid-lowering medications and to reduce residual inflammatory ASCVD risk in humans. In addition, further studies are needed to fully explore the fate of monocyte subsets in both mice and humans under stress. When taken together, these data provide important insights into the crucial role of chronic stress in CVD, highlighting that this common risk factor impairs the anti-atherosclerotic benefits of lipid-lowering medications and may represent an important determinant of residual ASCVD risk.

## MATERIAL AND METHODS

### Mice

Male C57BL/6 mice and B6.SJL-Ptprca Pepcb/BoyJ (Stock #: 002014, B6 CD45.1) were purchased from Jackson Laboratory. For standard chronic defeat stress (CSDS), 4–6-month-old male retired CD-1 breeders (Charles River Laboratories) were used as aggressors. Mice were allowed to habituate to the animal facility for at least 1 week after purchasing from external vendors. Aggressors were singly housed all times other than during the social defeats. All other animals were group housed before social defeat and paired housed following social defeat with a separating barrier. CSDS experiments were approved by the Animal Review Committee at the Icahn School of Medicine at Mount Sinai (IACUC 2014–0081 and IACUC LA12–00051) and were following relevant ethical regulations.

Mice were euthanized at the end of each experiment and perfused with saline containing 10% sucrose after blood collection via cardiac puncture. The hearts were removed at the proximal aorta, placed into a tissue mold, covered with OCT (optimal cutting temperature compound; Tissue-Tek), frozen in dry ice. Spleen, brown adipose and liver tissues were weighed and frozen immediately in liquid nitrogen, and stored at −80 °C. All animal experiments were performed according to protocols approved by the Institutional Animal Care Use Committee of New York University Grossman School of Medicine (PROTO201900104, IA16–00494 and PROTO202200120) and the Icahn School of Medicine (IACUC LA12–00051).

### Atherosclerosis regression study

C57BL/6 mice were injected with Pcsk9-AAV8 (AAV.8TBGmPCSK9D377Y, under the regulation of a liver-specific promoter; Penn Vector Core), at 1 × 10^12^ viral particles/mouse to promote hypercholesterolemia, as previously reported^1^ and fed with atherogenic diet (Research Diet: D18051006i: containing 10 % fat, 20 % protein, 70 % carbohydrate and 0.15 % cholesterol) for 24 weeks^2^ to induce atherosclerosis in the absence of obesity so eliminating obesity related metabolic syndrome. A subset of mice was used for baseline measurements, and the remainder were maintained on the same diet but containing MTP inhibitor (25 ppm Lomitapide, Cayman) to reduce cholesterol secretion from liver and induce atherosclerosis regression^3^. Before regression started, CSDS protocol was applied, and mice were grouped according to their social interaction test scores.

### Bone marrow transplantation (BMT) study

10 weeks-old B6.SJL-Ptprca Pepcb/BoyJ (Stock: 002014, B6 D45.1 from Jackson Laboratory) were used as bone marrow donors. C57BL/6 CD45.2 mice were lethally irradiated with a split dose of 2 × 600 cGy with an interval of 4 h between doses. BM cells (1×10^6^) were harvested from femora and tibias of donor control, stress susceptible and resilient mice and were introduced through a retro-orbital injection into recipients. The degree of repopulation by donor was determined by flow cytometry of CD45.1. After transplantation, host mice were treated with antibiotics (sulfatrim) for 4 weeks and given a minimum of 5 weeks. recovery to allow new immune cells to mature. Then, mice were injected intraperitoneally with *Pcsk9*-AAV8 (as above) and fed with atherogenic diet (D18051006i) for 24 weeks. The level of chimerism was assessed using flow cytometry, comparing CD45.1 (host) (mouse anti-CD45.1- PE/Dazzle^™^ 594, clone A20, Biolegend, 110747) and CD45.2 (donor) (mouse anti-CD45.2-BV421, clone 104, Biolegend, 109831) leukocytes.

### Chronic social defeat stress (CSDS)

For CSDS (previously described^4^), retired male CD-1 breeders (age: 4–6 months) were used as aggressors. Before each defeat, aggressors were screened for aggressive behavior for three consecutive days. Two days before the start of the defeat, CD-1 mice were housed on one side of a perforated Plexiglas partition. During 10 consecutive days of CSDS, experimental mice (7–8-week-old) were subjected to direct physical interaction with a CD-1 aggressor for 10 min per day (5 min for bone marrow chimera cohorts), and the rest of the day placed on the other side of the Plexiglas divider, allowing for sensory but not direct physical contact. Unstressed control mice were pair-housed across a Plexiglas partition. After the last day of defeat, stressed and unstressed control mice were paired housed across a Plexiglas partition. All stressed mice were carefully examined for wounding during the CSDS experiments and mice with excessive wounding were excluded.

#### Social interaction (SI) test.

SI testing was performed 24 h after the last defeat session under red light conditions. After a 1 h habituation period to the behavioral suite, mice were placed into a Plexiglas arena (42 cm × 42 cm × 42 cm, Nationwide Plastics) with a small, meshed enclosure on one end. For the first 2.5 min, the experimental mouse freely explored the arena. The mouse was then removed from the arena, which was subsequently cleaned with 70 % ethanol, then, a novel social target (CD-1 mouse) was placed into the enclosure and the experimental mouse was placed back into the arena for another 2.5 min. Locomotor activity was tracked and recorded using a Noldus Ethovision System (Noldus Information Technology Inc, Version 11.0, Leesburg, VA). SI ratio was calculated as the ratio between the time the experimental mouse spent in the vicinity of the enclosure (SI zone) when a target mouse was present vs. absent. Mice with an SI ratio of ≥ 1 show a behavioral profile like unstressed control mice and were termed resilient, while mice with an SI ratio < 1 were termed susceptible. Additional parameters that were measured were total locomotion and time spent in corners, calculated as the sum between the two corners opposite the wire enclosure.

### Aortic sinus sectioning and immunohistochemistry

Hearts after perfusion were embedded in OCT (Sakura, 4583: Torrance, CA) then frozen immediately and kept in −80 °C. Aortic root sections (6 mm) were collected via cryosectioning. For macrophage analysis, 18 sections 36 μm apart were stained with anti-CD68 antibody (Bio-Rad MCA 1957) and followed by VECTASTAIN ABC Alkaline Phosphatase Kit (Vector Laboratories) and the Vector Red Substrate Kit (Vector Laboratories) for visualization of antibody reactivity. Hematoxylin and Eosin staining were applied for morphometric analysis of lesion. Mean lesion area and necrotic core area (hematoxylin and eosin negative, acellular areas) were quantified using Image-Pro Plus software. Collagen content of the lesions was determined with Picrosirius red (PolyScience 24901–500: Niles, IL) and imaged under polarized light using a Keyence microscope.

### Monocyte trafficking assays in atherosclerosis

Monocytes were labeled as previously described^5^. 1 μm Fluoresbrite FITC-dyed (Yellow Green) plain microspheres (Polysciences Inc.) were diluted in PBS (1:4), and 250 μl was injected retro-orbitally into mice. Their incorporation efficiency in blood monocytes was assessed by flow cytometry, 24 hours after injection. Number of beads/lesions was counted as described^5^. For assessing trafficking of Ly6C^hi^ monocytes, mice were injected intraperitoneally with 1 mg/mouse EdU (Thermo Scientific, E10187). EdU labelling of circulating monocytes was detected using Click-iT^™^ EdU Cell Proliferation Kit, (Invitrogen, C10337) and in plaques with Alexa Fluor 647 nm Azide (Click-iT EdU Imaging Kit, Invitrogen) according to the manufacturer’s instructions. Sections were also stained with Ki67 (Abcam, 1666) and macrophage marker CD68 to detect proliferating macrophages in plaques. Slides were imaged using a Keyence microscope.

### Flow cytometry

#### Blood Leukocytes:

Freshly collected blood from tail and cardiac puncture were analyzed by using hematology cell counter (Heska Element HT-5) before flow cytometry. After red blood cell lysis, Single-cell suspensions were stained in FACS buffer. The following monoclonal antibodies were used for flow cytometry analyses at a dilution of 1/100: mouse anti- CD115 (CSF-1R) - PE, clone AFS98, Biolegend, 135506, mouse anti- Ly-6G- BV605, clone 1A8, Biolegend, 127639, mouse anti- Ly-6C-APC, clone HK1.4, Biolegend, 128016, mouse anti-CD45- PE/Cy7, clone 30-F11, BioLegend, 103114.

#### Hematopoietic stem and progenitor cells:

Bone marrow cells were collected from femurs and tibia after red blood cell lysis were counted and stained with a green live/dead stain (Invitrogen, L34969) for 30 min in 4 °C in the dark. Antibody cocktail for progenitor staining was added after washing. From eBioscience (San Diego, CA)- FITC anti-GR1 (11–5931-82), FITC anti-CD3 (11–0031-82), FITC anti-CD4 (11–0041-82), FITC anti-CD8 (11–0081-82), FITC anti-Ter119 (11–5921-82), FITC anti-CD19 (11–0193-82), FITC anti-NK1.1 (115941–82), FITC anf-CD2 (11–0021-82), APC anti-CD34 (50–0341-82), and from BioLegend (San Diego, CA)- FITC anti-CD11b (101206), PE-Cy7 anti-Sca1 (108114), APC-Cy7 anti-cKit (105826), PE anti-CD135 (135306), PerCP-Cy5.5 anti-CD150 (115922), BV605 antiCD48 (103441) and BV711 anti-CD16/32 (101337). Cells were kept at 4 °C, in the dark, overnight, then washed and analyzed using an LSRII flow cytometer (BD Biosciences; Franklin Lakes, NJ).

### Plaque macrophage profiling/laser capture microdissection (LCM)

A Leica DM6000 B instrument and Leica LMD CC7000 camera (Leica Microsystems) were used for LCM following the previously described protocol^6^. Staining was performed in an RNase-free environment. Serially sectioned guide slides that were stained with CD68 macrophage marker were prepared for each mouse and CD68^+^ areas in serial sections were collected by LCM. By using the Arcturus PicoPure RNA Isolation Kit (Life Technologies), RNA was extracted from CD68^+^ plaque macrophage. After RNA quality control and quantification, they were provided to NYU Genomics Core for Bulk RNA sequencing.

### Isolation and treatment of bone marrow cells

Bone marrow cells were isolated by flushing marrow from femurs and tibias, then plated in 96-well flat-bottom culture plates at a density of 270,000 cells per well in complete DMEM. After a 2-hour incubation period at 37 °C with 5 % CO₂, the cells were stimulated for 16 h with 100 ng/mL LPS or Pam3CSK4 or left untreated as a control. Following stimulation, supernatants were collected for cytokine analysis using ELISA.

### IL6 ELISA

ELISA plates were coated with purified (Biolegend, 504502, San Diego, CA) following the manufacturer’s instructions and incubated overnight at 4 °C. The plates were then washed four times with PBST (PBS containing 0.05 % Tween) and blocked with 1 % BSA in PBS for 1 h at room temperature. After blocking, the medium collected from bone marrow cells was added and incubated overnight at 4 °C. The following day, the plates were washed four times with PBST, and a biotinylated IL-6 antibody (Biolegend, 504602), as specified by the manufacturer, was added and incubated for 1 h at room temperature. After additional washes, alkaline phosphatase conjugated to streptavidin (Jackson ImmunoResearch, 016050084; West Grove, PA) was added per the manufacturer’s protocol and incubated for 30 min at room temperature. The plates were washed again, and alkaline phosphatase substrate (Sigma Aldrich, S0942; St. Louis, MO) was added. Finally, the plates were analyzed using a plate reader at 405 nm, and standard curves were generated using mouse recombinant IL-6 (PeproTech, 216–16; Cranbury, NJ).

### Cholesterol measurements

Total cholesterol was measured using Total cholesterol E kit (Wako Life Science, NC9138103).

### Human subjects

We collected data from a double-blind, randomized study conducted between August 2008 and December 2009 (NCT00703261), which analyzed the reduction of arterial inflammation in patients receiving statin therapy^7^. Inclusion and exclusion criteria were described previously^7^. We further selected patients for whom ^18^F-FDG uptake of the amygdala and temporal lobe was measurable. For each patient, we assessed stress-related neural activity (SNA) at baseline, expressed as the ratio between amygdalae and temporal lobe ^18^F-FDG uptake (amygdala to temporal lobe activity ratio: AmygA_C_). Arterial ^18^F-FDG uptake was evaluated at baseline and at 12-week follow-up, as previously described^7^. Briefly, arterial inflammation was defined as the average of the maximum target-to-background (TBR) activity, as the ratio of arterial to mean background venous blood pool activity within the most diseased segment of the index vessel (mean MDS TBR). The index vessel was identified as the artery with the highest ^18^F-FDG uptake at baseline among the carotids (right and left) and ascending aorta. High sensitivity C-reactive protein (hs-CRP) was measured at baseline and 12-week follow-up. Statistical analyses were performed using the Statistical Package for Social Sciences (SPSS; version 28, IBM Corporation, Armonk, NY, USA). Data were presented as mean and standard deviation (SD) if normally distributed or as the median and interquartile range (IQR) if skewed. Continuous variables were compared using independent sample t-tests or Mann-Whitney U test, as appropriate. In contrast, a one-way ANOVA test or Kruskall-Wallis test was used for analyses with more than two groups. Categorical variables were reported as absolute numbers and percentages and compared using Chi-square or Fisher’s exact tests, as appropriate. Multivariable linear regression was employed to assess the relationships between AmygA_C_, statin dose, and changes in arterial inflammation and hs-CRP.

### RNA sequencing

#### Bulk RNAseq analysis

Quality control of bulk sequencing reads was assessed using FastQC v0.11.7. Reads were first mapped to mouse reference genome mm10 using STAR v2.6.1d^8^, and gene counts were generated using the feature Counts function within Subread v1.6.3^9^. We then used R package DESeq2 v1.34.0^10^ to detect differential expression between the control, susceptible, and resilient groups. Unannotated transcripts (labeled with BAC clone IDs RP23- and RP24-) were removed. In each comparison, gene set enrichment analysis (GSEA) of Molecular Signatures Database GO, KEGG, and Hallmark gene sets was performed on upregulated genes with nominal p-value < 0.05 using the Bioconductor package clusterProfiler 4.2.2^11,12^

#### Single-cell RNA sequencing

Single cell RNA sequencing was performed on CD45^+^ cells isolated from bone marrows as described above. Bone marrow cells were pooled from 2 mice and stained with hashtagging antibodies and after washing they were stained for CD45 (anti-CD45, Biolegend) and live/dead cell staining with blue reactive dye. Single cell suspensions were isolated using the BD FACS Aria (BD bioscience, San Jose, USA).

During cell sorting, cellular debris was excluded with FSC and SSC gating. After cell sorting all groups were merged and loaded into single-cell gel beads (GEMs). They were barcoded with a unique molecular identifier using Single-Cell 3’ reagent kit (10X Genomics) and processes described previously^13^.

Hashtagging Antibodies:

**Table:** 

Bio legend	Barcode Sequence
TotalSeq^™^-B0301 anti-mouse Hashtag 1 Antibody	ACCCACCAGTAAGAC
TotalSeq^™^-B0302 anti-mouse Hashtag 2 Antibody	GGTCGAGAGCATTCA
TotalSeq^™^-B0303 anti-mouse Hashtag 3 Antibody	CTTGCCGCATGTCAT
TotalSeq^™^-B0304 anti-mouse Hashtag 4 Antibody	AAAGCATTCTTCACG
TotalSeq^™^-B0306 anti-mouse Hashtag 6 Antibody	TATGCTGCCACGGTA
TotalSeq^™^-B0307 anti-mouse Hashtag 7 Antibody	GAGTCTGCCAGTATC

#### Single-cell RNAseq analysis

Single cell RNAseq data was aligned to the mouse reference genome mm10 with Cell Ranger Single Cell Software Suite v6.0.1 to produce filtered gene-count matrices per sample, which were then analyzed using R package Seurat v4.0.3^14^. Cells with high outliers for number of UMIs (> 20k), number of genes (> 4.5k), or high mitochondrial gene expression (> 6%) were filtered out. The dataset was then normalized and demultiplexed into individual hashtag oligo samples. Gene expression matrix was normalized using the SCTransform function, followed by RunPCA and RunUMAP on the top 30 principal components. FindNeighbors and FindClusters functions were used for cell clustering, which identified 12 clusters with the Louvain algorithm. We then used R package SingleR, which leverages the ImmGen database to characterize cells by their closest match^15^. Pseudobulk method in conjunction with R package DEseq2 was then used to identify differentially expressed genes on monocytes across treatments (SUS vs CON, SUS vs RES, RES vs CON) followed by gene set enrichment analysis (GSEA) of Molecular Signatures Database GO, KEGG, and Hallmark gene sets on genes with nominal p-value < 0.05 using Bioconductor package clusterProfiler 4.2.2. Next, monocyte cells were subclustered using the same approach as above, identifying 5 subclusters. The FindAllMarkers function was applied to determine markers for each subcluster, and function enrichGO from clusterProfiler was used to perform pathway enrichment analysis on markers from clusters of interest 0 and 2.

AUCell^16^ was used to calculate cell-wise activity scores of pathways in the MSigDB hallmark gene set collection^17^. A Wilcoxon test was used to test differences in activity scores between treatment groups. P-values were adjusted for multiple testing using Bonferroni correction.

Activity scores of regulons (transcription factors and their predicted target genes) were estimated using pySCENIC^18^. Using the R package Seurat^19^, regulon activity scores were scaled and UMAP dimension reduction was performed using the first 10 principal components. A Wilcoxon test in Seurat’s Find Markers function was used to test differences in regulon activity between treatment groups, and to obtain fold changes and Bonferroni adjusted p-values.

All analyses in R were performed in R v4.2.2 (R Core Team 2021). Figures were made using the R packages ggplot2^20^ and ComplexHeatmap^21^.

### Statistical analysis

GraphPad Prism software (Version 10, GraphPad Software Inc.) was used for statistical analysis. Outliers were identified using the Grubbs’s test and excluded from statistical analyses. The level of statistical significance was set at p < 0.05. For multiple comparisons, non-parametric multiple-comparison tests comparing the mean rank of each group (when Gaussian distribution was not assumed) or one or two-way ANOVA followed by Dunnett multiple-comparisons test for one-way ANOVA and Sidak multiple-comparisons test for two-way ANOVA. The unpaired two-tailed Student’s t-test was used for two groups comparison.

## Supplementary Files

Supplementary Information is available for this paper.

This is a list of supplementary files associated with this preprint. Click to download.

- EXTENDEDDATATABLESANDFIGURES.docx

## Data Availability

All the other data are available from the corresponding author upon reasonable request.

## References

[1] Kivimäki M. & Steptoe A. Effects of stress on the development and progression of cardiovascular disease. Nature Reviews Cardiology 15, 215–229 (2018). 10.1038/nrcardio.2017.189 29213140

[2] Vaccarino V. & Bremner J. D. Stress and cardiovascular disease: an update. Nature Reviews Cardiology (2024). 10.1038/s41569-024-01024-y PMC11872152 38698183

[3] Arnold S. V., Smolderen K. G., Buchanan D. M., Li Y. & Spertus J. A. Perceived stress in myocardial infarction: long-term mortality and health status outcomes. J Am Coll Cardiol 60, 1756–1763 (2012). 10.1016/j.jacc.2012.06.044 23040574 PMC3601381

[4] Kumric M. Emerging Therapies for the Treatment of Atherosclerotic Cardiovascular Disease: From Bench to Bedside. Int J Mol Sci 24 (2023). 10.3390/ijms24098062 PMC10178593 37175766

[5] Williams K. J., Feig J. E. & Fisher E. A. Rapid regression of atherosclerosis: insights from the clinical and experimental literature. Nature Clinical Practice Cardiovascular Medicine 5, 91–102 (2008). 10.1038/ncpcardio1086 18223541

[6] Sarraju A. & Nissen S. E. Atherosclerotic plaque stabilization and regression: a review of clinical evidence. Nature Reviews Cardiology 21, 487–497 (2024). 10.1038/s41569-023-00979-8 38177454

[7] Ridker P. M. How Common Is Residual Inflammatory Risk? Circ Res 120, 617–619 (2017). 10.1161/circresaha.116.310527 28209792

[8] Heidt T. Chronic variable stress activates hematopoietic stem cells. Nat Med 20, 754–758(2014). 10.1038/nm.3589 24952646 PMC4087061

[9] Giannarelli C. Susceptibility to chronic social stress increases plaque progression, vulnerability and platelet activation. Thromb Haemost 117, 816–818 (2017). 10.1160/th16-10-0817 28078352 PMC5490439

[10] Hinterdobler J. Acute mental stress drives vascular inflammation and promotes plaque destabilization in mouse atherosclerosis. Eur Heart J 42, 4077–4088 (2021). 10.1093/eurheartj/ehab371 34279021 PMC8516477

[11] Osborne M. T. Disentangling the Links Between Psychosocial Stress and Cardiovascular Disease. Circulation: Cardiovascular Imaging 13, e010931 (2020). 10.1161/CIRCIMAGING.120.010931 32791843 PMC7430065

[12] Barrett T. J. Chronic stress primes innate immune responses in mice and humans. Cell Rep 36, 109595 (2021). 10.1016/j.celrep.2021.109595 34496250 PMC8493594

[13] Powell N. D. Social stress up-regulates inflammatory gene expression in the leukocyte transcriptome via β-adrenergic induction of myelopoiesis. Proceedings of the National Academy of Sciences 110, 16574–16579 (2013). 10.1073/pnas.1310655110 PMC3799381 24062448

[14] Engler H., Bailey M. T., Engler A. & Sheridan J. F. Effects of repeated social stress on leukocyte distribution in bone marrow, peripheral blood and spleen. J Neuroimmunol 148, 106–115 (2004). 10.1016/j.jneuroim.2003.11.011 14975591

[15] Tawakol A. Relation between resting amygdalar activity and cardiovascular events: a longitudinal and cohort study. The Lancet 389, 834–845 (2017). 10.1016/S0140-6736(16)31714-7 PMC7864285 28088338

[16] Boukezzi S. Exaggerated amygdala response to threat and association with immune hyperactivity in depression. Brain Behav Immun 104, 205–212 (2022). 10.1016/j.bbi.2022.05.015 35636614 PMC10966680

[17] Biltz R. G. Antagonism of the brain P2X7 ion channel attenuates repeated social defeat induced microglia reactivity, monocyte recruitment and anxiety-like behavior in male mice. Brain Behav Immun 115, 356–373 (2024). 10.1016/j.bbi.2023.10.011 37914101 PMC10807695

[18] Munshi S. Repeated stress induces a pro-inflammatory state, increases amygdala neuronal and microglial activation, and causes anxiety in adult male rats. Brain Behav Immun 84, 180–199 (2020). 10.1016/j.bbi.2019.11.023 31785394 PMC7010555

[19] Kang D. O. Stress-associated neurobiological activity is linked with acute plaque instability via enhanced macrophage activity: a prospective serial 18F-FDG-PET/CT imaging assessment. Eur Heart J 42, 1883–1895 (2021). 10.1093/eurheartj/ehaa1095 33462618

[20] Cathomas F. Circulating myeloid-derived MMP8 in stress susceptibility and depression. Nature 626, 1108–1115 (2024). 10.1038/s41586-023-07015-2 38326622 PMC10901735

[21] Menard C. Social stress induces neurovascular pathology promoting depression. Nat Neurosci 20, 1752–1760 (2017). 10.1038/s41593-017-0010-3 29184215 PMC5726568

[22] Hodes G. E. Individual differences in the peripheral immune system promote resilience versus susceptibility to social stress. Proc Natl Acad Sci U S A 111, 16136–16141 (2014). 10.1073/pnas.1415191111 25331895 PMC4234602

[23] Peled M. A wild-type mouse-based model for the regression of inflammation in atherosclerosis. PLoS One 12, e0173975 (2017). 10.1371/journal.pone.0173975 28291840 PMC5349694

[24] Yu H. Atherosclerotic Plaque Regression: Experimental Approaches and Therapeutic Advances. Trends in Cell Biology 31, 424–427 (2021). 10.1016/j.tcb.2021.03.003 33726967

[25] Shapiro M. D., Tavori H. & Fazio S. PCSK9. Circulation Research 122, 1420–1438 (2018). 10.1161/CIRCRESAHA.118.311227 29748367 PMC5976255

[26] Bjørklund M. M. Induction of Atherosclerosis in Mice and Hamsters Without Germline Genetic Engineering. Circulation Research 114, 1684–1689 (2014). 10.1161/CIRCRESAHA.114.302937 24677271

[27] Hewing B. Rapid regression of atherosclerosis with MTP inhibitor treatment. Atherosclerosis 227, 125–129 (2013). 10.1016/j.atherosclerosis.2012.12.026 23332773 PMC4047651

[28] Golden S. A., Covington H. E., Berton O. & Russo S. J. A standardized protocol for repeated social defeat stress in mice. Nature Protocols 6, 1183–1191 (2011). 10.1038/nprot.2011.361 21799487 PMC3220278

[29] Krishnan V. Molecular Adaptations Underlying Susceptibility and Resistance to Social Defeat in Brain Reward Regions. Cell 131, 391–404 (2007). 10.1016/j.cell.2007.09.018 17956738

[30] Li L. Social trauma engages lateral septum circuitry to occlude social reward. Nature 613, 696–703 (2023). 10.1038/s41586-022-05484-5 36450985 PMC9876792

[31] Chistiakov D. A., Killingsworth M. C., Myasoedova V. A., Orekhov A. N. & Bobryshev Y. V. CD68/macrosialin: not just a histochemical marker. Laboratory Investigation 97, 4–13 (2017). 10.1038/labinvest.2016.116 27869795

[32] Fuster V. Lewis A. Conner Memorial Lecture. Mechanisms leading to myocardial infarction: insights from studies of vascular biology. Circulation 90, 2126–2146 (1994). 10.1161/01.CIR.90.4.2126 7718033

[33] Li Y. H. Occurrences and Functions of Ly6C(hi) and Ly6C(lo) Macrophages in Health and Disease. Front Immunol 13, 901672 (2022). 10.3389/fimmu.2022.901672 35707538 PMC9189283

[34] Weinstock A. & Fisher E. A. Methods to Study Monocyte and Macrophage Trafficking in Atherosclerosis Progression and Resolution. Methods Mol Biol 1951, 153–165 (2019). 10.1007/978-1-4939-9130-3_12 30825151 PMC7075492

[35] Robbins C. S. Local proliferation dominates lesional macrophage accumulation in atherosclerosis. Nat Med 19, 1166–1172 (2013). 10.1038/nm.3258 23933982 PMC3769444

[36] Feig J. E. & Fisher E. A. Laser capture microdissection for analysis of macrophage gene expression from atherosclerotic lesions. Methods Mol Biol 1027, 123–135 (2013). 10.1007/978-1-60327-369-5_5 23912984 PMC4278963

[37] Wong K. L. Gene expression profiling reveals the defining features of the classical, intermediate, and nonclassical human monocyte subsets. Blood 118, e16–e31 (2011). 10.1182/blood-2010-12-326355 21653326

[38] Zenz R. Activator protein 1 (Fos/Jun) functions in inflammatory bone and skin disease. Arthritis Research & Therapy 10, 201 (2008). 10.1186/ar2338 18226189 PMC2374460

[39] Drechsler M., Duchene J. & Soehnlein O. Chemokines control mobilization, recruitment, and fate of monocytes in atherosclerosis. Arterioscler Thromb Vasc Biol 35, 1050–1055 (2015). 10.1161/ATVBAHA.114.304649 25792446

[40] Teh Y. C., Ding J. L., Ng L. G. & Chong S. Z. Capturing the Fantastic Voyage of Monocytes Through Time and Space. Front Immunol 10, 834 (2019). 10.3389/fimmu.2019.00834 31040854 PMC6476989

[41] Thomas G., Tacke R., Hedrick C. C. & Hanna R. N. Nonclassical Patrolling Monocyte Function in the Vasculature. Arteriosclerosis, Thrombosis, and Vascular Biology 35, 1306–1316 (2015). 10.1161/ATVBAHA.114.304650 25838429 PMC4441550

[42] Ma R. Y., Black A. & Qian B. Z. Macrophage diversity in cancer revisited in the era of single-cell omics. Trends Immunol 43, 546–563 (2022). 10.1016/j.it.2022.04.008 35690521

[43] Miyake K. Single-cell transcriptomics identifies the differentiation trajectory from inflammatory monocytes to pro-resolving macrophages in a mouse skin allergy model. Nature Communications 15, 1666 (2024). 10.1038/s41467-024-46148-4 PMC10891131 38396021

[44] Ramachandran P. Differential Ly-6C expression identifies the recruited macrophage phenotype, which orchestrates the regression of murine liver fibrosis. Proc Natl Acad Sci U S A 109, E3186–3195 (2012). 10.1073/pnas.1119964109 23100531 PMC3503234

[45] Yurdagul A. Jr., Doran A. C., Cai B., Fredman G. & Tabas I. A. Mechanisms and Consequences of Defective Efferocytosis in Atherosclerosis. Front Cardiovasc Med 4, 86 (2017). 10.3389/fcvm.2017.00086 29379788 PMC5770804

[46] Mildner A. Genomic Characterization of Murine Monocytes Reveals C/EBPβ Transcription Factor Dependence of Ly6C– Cells. Immunity 46, 849–862.e847 (2017). 10.1016/j.immuni.2017.04.018 28514690

[47] Kumar N., Mishra B., Athar M. & Mukhtar S. Inference of Gene Regulatory Network from Single-Cell Transcriptomic Data Using pySCENIC. Methods Mol Biol 2328, 171–182 (2021). 10.1007/978-1-0716-1534-8_10 34251625

[48] Fowler T., Sen R. & Roy Ananda L. Regulation of Primary Response Genes. Molecular Cell 44, 348–360 (2011). 10.1016/j.molcel.2011.09.014 22055182 PMC3212756

[49] Bahrami S. & Drabløs F. Gene regulation in the immediate-early response process. Advances in Biological Regulation 62, 37–49 (2016). 10.1016/j.jbior.2016.05.001 27220739

[50] Bruns H. the IKZF1-IRF4 Axis Regulates Macrophage Polarization and Macrophage-Mediated Anti-Tumor Immunity. Blood 128, 2514–2514 (2016). 10.1182/blood.V128.22.2514.2514

[51] O’Connor K. W. *Bcl6*, *Irf2*, and *Notch2* promote nonclassical monocyte development. Proceedings of the National Academy of Sciences 120, e2220853120 (2023). 10.1073/pnas.2220853120 PMC10469339 37607223

[52] Medrano J. L. & Naya F. J. The transcription factor MEF2A fine-tunes gene expression in the atrial and ventricular chambers of the adult heart. J Biol Chem 292, 20975–20988 (2017). 10.1074/jbc.M117.806422 29054930 PMC5743072

[53] Gamrekelashvili J. Regulation of monocyte cell fate by blood vessels mediated by Notch signalling. Nat Commun 7, 12597 (2016). 10.1038/ncomms12597 27576369 PMC5013671

[54] Wang J., Liu Y. M., Hu J. & Chen C. Trained immunity in monocyte/macrophage: Novel mechanism of phytochemicals in the treatment of atherosclerotic cardiovascular disease. Front Pharmacol 14, 1109576 (2023). 10.3389/fphar.2023.1109576 36895942 PMC9989041

[55] Rahman K. Inflammatory Ly6Chi monocytes and their conversion to M2 macrophages drive atherosclerosis regression. J Clin Invest 127, 2904–2915 (2017). 10.1172/JCI75005 28650342 PMC5531402

[56] Luan Y. Y. & Yao Y. M. The Clinical Significance and Potential Role of C-Reactive Protein in Chronic Inflammatory and Neurodegenerative Diseases. Front Immunol 9, 1302 (2018). 10.3389/fimmu.2018.01302 29951057 PMC6008573

[57] Dar T. Greater Neurobiological Resilience to Chronic Socioeconomic or Environmental Stressors Associates With Lower Risk for Cardiovascular Disease Events. Circ Cardiovasc Imaging 13, e010337 (2020). 10.1161/circimaging.119.010337 32787499 PMC7820711

[58] Janssen H., Koekkoek L. L. & Swirski F. K. Effects of lifestyle factors on leukocytes in cardiovascular health and disease. Nat Rev Cardiol 21, 157–169 (2024). 10.1038/s41569-023-00931-w 37752350

[59] Poller W. C. Brain motor and fear circuits regulate leukocytes during acute stress. Nature 607, 578–584 (2022). 10.1038/s41586-022-04890-z 35636458 PMC9798885

[60] Hodes G. E. Individual differences in the peripheral immune system promote resilience versus susceptibility to social stress. Proceedings of the National Academy of Sciences 111, 16136–16141 (2014). 10.1073/pnas.1415191111 PMC4234602 25331895

